# Modified FOLFIRINOX as a second-line therapy following gemcitabine plus nab-paclitaxel therapy in metastatic pancreatic cancer

**DOI:** 10.1186/s12885-020-06945-8

**Published:** 2020-05-20

**Authors:** Masashi Sawada, Akiyoshi Kasuga, Takafumi Mie, Takaaki Furukawa, Takanobu Taniguchi, Koshiro Fukuda, Yuto Yamada, Tsuyoshi Takeda, Ryo Kanata, Masato Matsuyama, Takashi Sasaki, Masato Ozaka, Naoki Sasahira

**Affiliations:** 1 Division of Hepato-Biliary-Pancreatic Medicine, Department of Gastroenterology, Cancer Institute Hospital of Japanese Foundation for Cancer Research, 3-8-31, Ariake, Koto, Tokyo, 135-8550 Japan; 2 Division of Gastroenterology and Hepatology, Department of Internal Medicine, School of Medicine, Keio University, 3-8-31, Ariake, Koto, Tokyo, 135-8550 Japan

**Keywords:** Pancreatic cancer, Second-line chemotherapy, Modified FOLFIRINOX, Gemcitabine, Nab-paclitaxel, Prognostic factor

## Abstract

**Background:**

There is no established second-line treatment after failure of gemcitabine plus nab-paclitaxel (GnP) therapy for metastatic pancreatic cancer (MPC). The purpose of this study was to evaluate the efficacy and tolerability of the modified FOLFIRINOX (mFFX) as a second-line therapy for MPC and to investigate prognostic factors for survival.

**Methods:**

From 2015 to 2019, we retrospectively reviewed the medical records of consecutive patients receiving mFFX for MPC after failure of GnP therapy. Patients were treated every 2 weeks with mFFX (intravenous oxaliplatin 85 mg/m^2^, intravenous irinotecan 150 mg/m^2^, and continuous infusion of 5-fluorouracil 2400 mg/m^2^ for 46 h without bolus infusion).

**Results:**

In total, 104 patients received mFFX. The median overall survival (OS) was 7.0 months (95% confidence interval [CI]: 6.2–9.8) and the progression-free survival (PFS) 3.9 months (95% CI 2.8–5.0). The objective response rate was 10.6% and the disease control rate 56.7%. The median relative dose intensities of oxaliplatin, irinotecan, and infusional 5-FU were 80.0% (range 21.5–100%), 77.2% (range 38.1–100%), and 85.9% (range 36.9–100%), respectively. Grade 3–4 toxicities were reported in 57 patients (54.8%), including neutropenia, leukopenia, anemia, febrile neutropenia, and peripheral sensory neuropathy. Glasgow prognostic score and carcinoembryonic antigen level were independently associated with survival. Our prognostic model using these parameters could classify the patients into good (*n* = 38), intermediate (*n* = 47), and poor (*n* = 19) prognostic groups. The median OS and PFS time was 14.7 (95% CI 7.6–16.3) and 7.6 months (95% CI 4.1–10.5) for the good prognostic factors, 6.5 (95% CI 5.5–10.0) and 3.6 months (95% CI 2.7–4.8) for the intermediate prognostic factors and 5.0 (95% CI 2.9–6.6) and 1.7 months (95% CI 0.9–4.3) for the poor prognostic factors, respectively.

**Conclusions:**

The mFFX showed to be a tolerable second-line treatment for MPC after GnP failure. Our prognostic model might be useful for deciding whether mFFX is indicated in this setting.

## Background

Pancreatic cancer (PC) is a lethal disease and is the fourth leading cause of cancer-related deaths in the United States and Europe and accounts for > 33,000 deaths in Japan annually [1]. Although multidisciplinary and multimodality treatment approaches are important in improving the survival of patients with PC, more than half of patients are diagnosed in the metastatic stage with dismal prognosis. Conventional cytotoxic chemotherapy remains the standard treatment for patients with metastatic pancreatic cancer (MPC).

Gemcitabine (GEM) monotherapy has long been the standard first-line chemotherapy for MPC [2]. Recently, a combination therapy of FOLFIRINOX (FFX) (5-fluorouracil [5-FU], leucovorin, irinotecan, and oxaliplatin) and GEM plus nab-paclitaxel (GnP) has shown superiority over GEM monotherapy [3, 4]. To reduce the severe toxicity of FFX, the use of a modified FFX (mFFX) as a first-line treatment has been evaluated and has demonstrated reduced number of adverse events and a similar efficacy to FFX [5]. This modified FFX regimen is widely accepted in daily practice in Japan. With the growing number of available drugs for PC, the overall treatment outcomes for MPC treated with chemotherapy are significantly improved in daily practice [6, 7].

Although several randomized clinical studies have shown survival benefits of second-line chemotherapy after GEM-based therapy, there is no established standard second-line chemotherapy regimen for MPC [8–10]. Moreover, both FFX and GnP have shown better outcomes than GEM alone. Sequential therapies based on FFX followed by GnP, or vice versa, have been tested and used in practice. However, there is limited research about mFFX after failure of GnP. This retrospective study aimed to evaluate the efficacy and tolerability of mFFX after failure of first-line GnP in patients with MPC and to clarify the characteristics of patients who will benefit from second-line mFFX.

## Methods

### Patients

Between April 2015 and March 2019, this study enrolled consecutive patients with MPC who received mFFX after GnP therapy at our hospital. We retrospectively reviewed the medical records of the patients from the prospectively maintained institutional database for PC.

The patient selection criteria were as follows: presence of a pathological and clinical diagnosis of metastatic adenocarcinoma; disease progression or intolerance while under GnP chemotherapy; an Eastern Cooperative Oncology Group performance status (ECOG PS) of 0–2; no prior chemotherapy with 5-FU, oxaliplatin, or irinotecan; and adequate bone marrow, renal, and liver functions.

The exclusion criteria were as follows: dose reduction of any drug from the first cycle with the exception of reduction of irinotecan due to a uridine diphosphate glucuronosyltransferase (UGT) 1A1 status as described below; locally advanced pancreatic carcinoma; and severe complications such as active infection, massive pleural effusion or ascites, uncontrolled diabetes, and active concomitant malignancy.

### Treatment

Patients were treated with mFFX every 2 weeks, as follows: a 2-h intravenous infusion of oxaliplatin 85 mg/m^2^, a 2-h intravenous infusion of l-leucovorin 200 mg/m^2^, a 90-min intravenous infusion of irinotecan 150 mg/m^2^, and a continuous 46-h intravenous infusion of 5-FU 2400 mg/m^2^, with an omission of bolus 5-FU infusion [5]. All patients routinely received palonosetron 0.75 mg intravenously, dexamethasone 6.6 mg intravenously, and aprepitant 125 mg orally on day 1, followed by aprepitant 80 mg orally on days 2 and 3 and dexamethasone 8 mg orally on days 2–4 for antiemetic prophylaxis. Granulocyte-colony stimulating factor was not allowed as the primary prophylaxis. Irinotecan was reduced to 100 mg/m^2^ for patients with UGT genetic polymorphisms such as homozygous UGT1A1*28 or UGT1A1*6 and heterozygous UGT1A1*6 or UGT1A1*28. For patients with no available results for UGT genetic polymorphism status, irinotecan was started with the initial dose reduced to 100 mg/m^2^. The dose of any drug was reduced at the discretion of the treating physician, according to the presence of adverse events. The treatment was continued until disease progression, unacceptable toxicity, or patient refusal.

### Data collection and evaluation

Pretreatment evaluation included collection of data on age, sex, ECOG PS, location of PC, disease status, metastatic sites (liver, lung, and peritoneum), and presence of biliary drainage. Laboratory variables such as carcinoembryonic antigen (CEA), carbohydrate antigen 19–9 (CA19–9), albumin (Alb), and C-reactive protein (CRP) levels and neutrophil and lymphocyte counts were initially recorded as continuous variables. Quantitative data were expressed as medians (with ranges), and qualitative data as percentages. The continuous variables were later dichotomized according to the median or reference value of each variable.

We evaluated the relative dose intensity (RDI) of oxaliplatin, irinotecan, and 5-FU. RDI was calculated as the ratio of the actual dose intensity (ADI) to the standard dose intensity (SDI), where ADI was the ratio of the actual dose to the actual duration of chemotherapy, and SDI was the ratio of the standard dose to the standard duration of the regimen. Tumor response was assessed every 2–3 months using contrast-enhanced computed tomography, according to the Response Evaluation Criteria in Solid Tumors (RECIST) version 1.1. Progression-free survival (PFS) was counted from the date of treatment initiation to the date of documentation of disease progression or the last follow-up, while overall survival (OS) was counted from the date of treatment initiation to the date of death or the last follow-up. OS and PFS were calculated using the Kaplan-Meier method. Adverse effects were graded using the National Cancer Institute Common Terminology Criteria for Adverse Events (CTCAE) version 5.0. As inflammation-based prognostic factors, Glasgow prognostic score (GPS) and neutrophil-to-lymphocyte ratio (NLR) were also evaluated [11, 12]. GPS was defined as follows: patients with both an elevated CRP level (> 1.0 mg/dL) and hypoalbuminemia (Alb < 3.5 g/dL) were allocated a score of 2; patients with only one of these biochemical abnormalities were given a score of 1; and patients with neither of these abnormalities were scored 0. Clinical data were monitored until May 2019.

### Statistical analysis

Relationships between survival and clinical variables were investigated using univariate and multivariate analyses. The log-rank test was used to evaluate differences in survival. The multivariate analysis was carried out using stepwise Cox proportional hazards regression modeling to identify independent prognostic factors. Each patient was then assigned a prognostic index value, calculated based on the number of major independent protnostic factors of survival and weighted using the likelihood ratio of the independent factors. Patients were stratified based on this prognostic index. *p* values of < 0.05 were considered statistically significant. All statistical analyses were performed using the SPSS statistical software program (version 20.0; SPSS, Chicago, IL, USA).

## Results

### Patient characteristics

The characteristics of the subjects are shown in Table 1. The study enrolled 104 patients. The median age was 63 years. Among the 104 patients, 75 (72.1%) were male, 103 (99.0%) had an ECOG PS of 0–1, 26.0% had the head of the pancreas as the primary tumor site, 29.8% experienced recurrence after resection, and 17.3% had a biliary stent. The major site of metastasis was the liver (69.2%). UGT genetic polymorphisms were classified as wild-type (46.2%), heterozygote (44.2%), double heterozygote (3.8%), and homozygote (3.8%), with some patients having no available data (1.9%). The median time to failure of GnP therapy was 194 days. Thirty-one (29.8%) patients were treated with pregabalin or duloxetine for peripheral sensory neuropathy (PN) induced by nab-paclitaxel. The mFFX therapy was usually started because of disease progression (97%) and intolerance (3%).

**Table 1:** Patient characteristics

Characteristic		%
Age (years)		
Median	63	
Range	37–77	
Sex		
Male	75	72.1
Female	29	27.9
ECOG PS		
0	93	89.4
1	10	9.6
2	1	1
Location of pancreatic tumor		
Head	27	26
Body/tail	46	44.2
Recurrent after resection	31	29.8
Metastatic site		
Liver	72	69.2
Lung	19	18.3
Peritoneum	16	15.4
Biliary drainage		
Yes	18	17.3
No	86	82.7
UGT1A1*6/UGT1A1*28		
Wild/wild	48	46.2
Wild/heterozygous	13	12.5
Heterozygous/wild	33	31.7
Heterozygous/heterozygous	4	3.8
Homozygous (*6 or *28)	4	3.8
NA	2	1.9
CEA (ng/mL)		
Median	6.1	
Range	1.2–10,068	
CA19–9 (IU/mL)		
Median	814.5	
Range	2–50,000	
Alb (g/dL)		
Median	3.6	
Range	2.5–4.4	
CRP (mg/dL)		
Median	0.31	
Range	0.01–8.75	

*Alb* albumin, *CA19–9* carbohydrate antigen 19–9, *CEA* carcinoembryonic antigen, *CRP* C-reactive protein, *ECOG PS* Eastern Cooperative Oncology Group performance status, *NA* not available, *UGT1A1* uridine diphosphate glucuronosyltransferase 1A1

### Toxicity

Adverse events are summarized in Table 2. Grade 3–4 toxicities occurred in 57 patients (54.8%). The major grade 3–4 hematological toxicities were neutropenia (42.3%), leucopenia (24.0%), and anemia (17.3%). The incidence of febrile neutropenia was 5.8%. The major grade 3–4 nonhematological toxicities were PN (10.6%), diarrhea (2.9%), and anorexia (1.0%). Interstitial pneumonia occurred in one patient (1.0%). There were no treatment-related deaths.

**Table 2:** Adverse events according to the Common Terminology Criteria for Adverse Events (CTCAE) version 5.0

Toxicity	All grades		Grade 3 or higher	
Hematologic				
Anemia	101	(97.1%)	18	(17.3%)
Neutropenia	69	(66.3%)	44	(42.3%)
Leukopenia	69	(66.3%)	25	(24.0%)
Thrombocytopenia	73	(70.2%)	2	(1.9%)
Nonhematologic				
Febrile neutropenia			6	(5.8%)
Anorexia	43	(41.3%)	1	(1.0%)
Fatigue	89	(85.6%)	0	(0.0%)
Stomatitis	32	(30.8%)	0	(0.0%)
Diarrhea	63	(60.6%)	3	(2.9%)
Constipation	50	(48.1%)	0	(0.0%)
Nausea	71	(68.3%)	0	(0.0%)
PN	93	(89.4%)	11	(10.6%)
Edema in limbs	29	(27.9%)	0	(0.0%)

*PN* peripheral sensory neuropathy

### Treatment exposure

The median follow-up time was 188 days (range 14–707). A total of 883 cycles were delivered to the 104 patients. The median number of treatment cycles was 6 (range 1–47). The median RDIs of oxaliplatin, irinotecan, and infusional 5-FU were 80.0% (range 21.5–100%), 77.2% (range 38.1–100%), and 85.9% (range 36.9–100%), respectively. Dose reduction occurred in 75 patients (72.1%), and treatment delay in 71 patients (68.2%). Neutropenia was the most frequent cause of both dose reduction and treatment delay. Oxaliplatin treatment was suspended in 18 patients (17.3%). The reasons for treatment suspension were PN in 10 patients (79.8%), allergy in 3 (2.9%), and others in 5. The median number of cycles of mFFX before the suspension of oxaliplatin due to PN was 12 (range 3–18). On the other hand, the reasons for treatment discontinuation were disease progression in 83 patients (79.8%), adverse events in 2 (1.9%), and transfer to another hospital in 2 (1.9%). Thus, 17 patients were on treatment.

### Efficacy

**Fig. 1:**
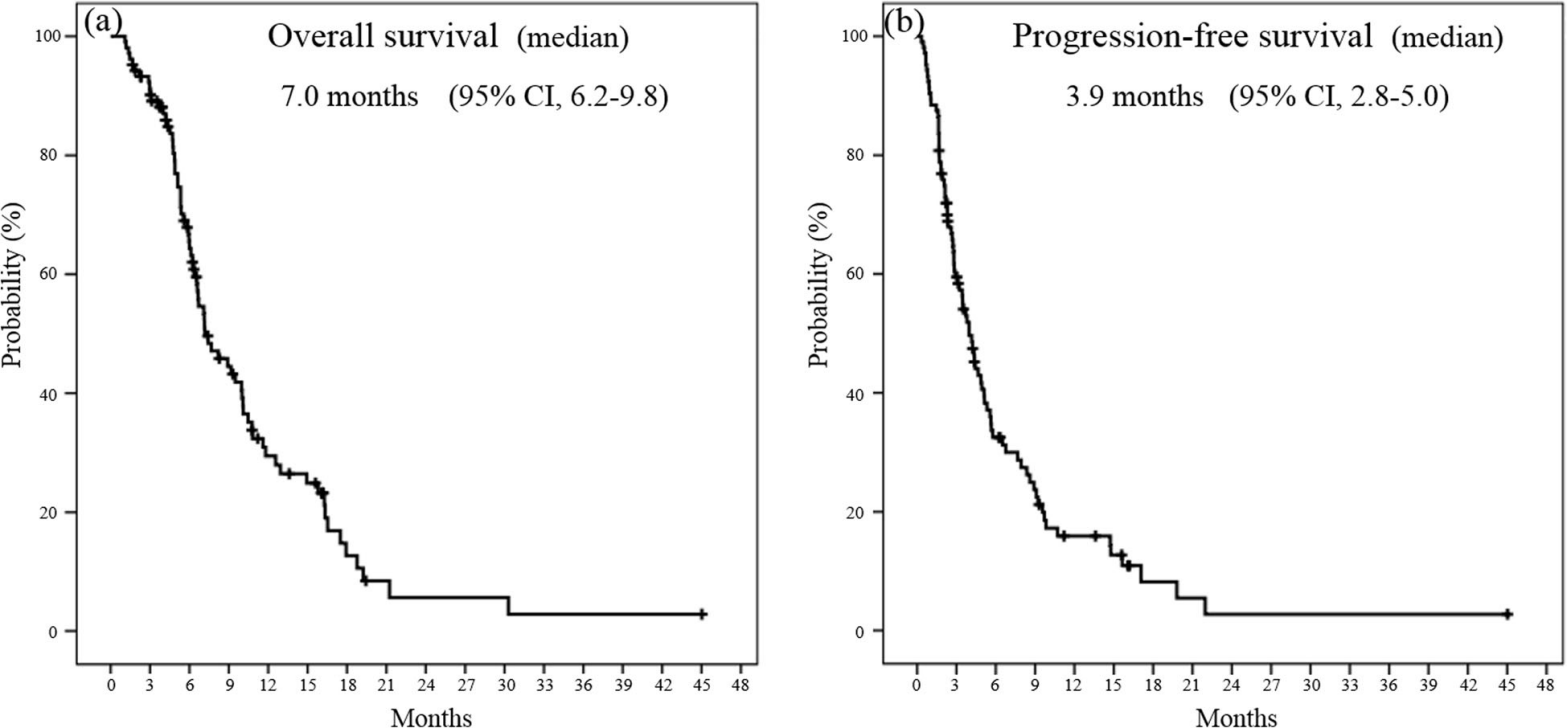
Kaplan-Meier analysis of (**a**) overall survival and (**b**) progression-free survival for all patients treated with modified FOLFIRINOX as a second-line therapy following gemcitabine plus nab-paclitaxel. *CI* confidence interval

Eleven (10.6%) patients showed partial responses, while 48 (46.2%) showed stable disease, resulting in a disease control rate of 56.7%. The median OS time was 7.0 months (95% confidence interval [CI] 6.2–9.8), and the median PFS time was 3.9 months (95% CI 2.8–5.0) (Fig. 1). The median OS time after the start of the first-line therapy was 15.9 months (95% CI 13.5–18.8).

After disease progression on mFFX therapy, 34 patients (32.7%) received a third line of chemotherapy, as follows: 14 patients (13.5%) received GEM plus erlotinib, 13 (12.5%) received S-1 monotherapy, and 7 (6.7%) received other regimens.

### Prognostic factors

The median survival time and *p* values for the univariate analysis are shown in Table 3. Among the variables, ECOG PS = 0, CEA level ≤ 10 ng/mL, Alb ≥3.5 g/dL, NLR ≤ 5.0, CRP ≤ 0.16 g/dL, and GPS = 0 were significantly associated with longer survival. CA19–9 level was not a prognostic factor.

**Table 3:** Univariate analysis of prognostic factors

Prognostic factor	n	Median OS (months)	*p* value
Sex			
Male	75	6.5	0.052
Female	29	10.6	
ECOG PS			
0	93	7.3	0.038
1, 2	11	5.2	
Age			
≥ 70	12	7	0.334
< 70	92	7.3	
Pancreatic resection			
Yes	31	7	0.319
No	73	8	
First-line therapy duration			
≤ 4 months	19	7	0.459
> 4 months	85	7.3	
Liver metastasis			
Yes	72	6.5	0.054
No	32	9.9	
CEA (ng/mL)			
≤ 10	69	9.9	< 0.001
> 10	35	6.2	
CA19–9 (IU/mL)			
≤ 1000	57	7.5	0.621
> 1000	47	7	
Alb (g/dL)			
< 3.5	40	6	0.014
≥ 3.5	64	9.4	
CRP (mg/dL)			
≤ 0.16	35	11.6	< 0.001
> 0.16	69	5.9	
GPS			
Low (0)	44	10.6	< 0.001
High (1, 2)	60	6	
NLR			
≤ 5.0	96	8.7	< 0.001
> 5.0	8	4.6	

*Alb* albumin, *CA19–9* carbohydrate antigen 19–9, *CEA* carcinoembryonic antigen, *CRP* C-reactive protein, *ECOG PS* Eastern Cooperative Oncology Group performance status, *GPS* Glasgow prognostic score, *NLR* neutrophil-to-lymphocyte ratio

**Fig. 2:**
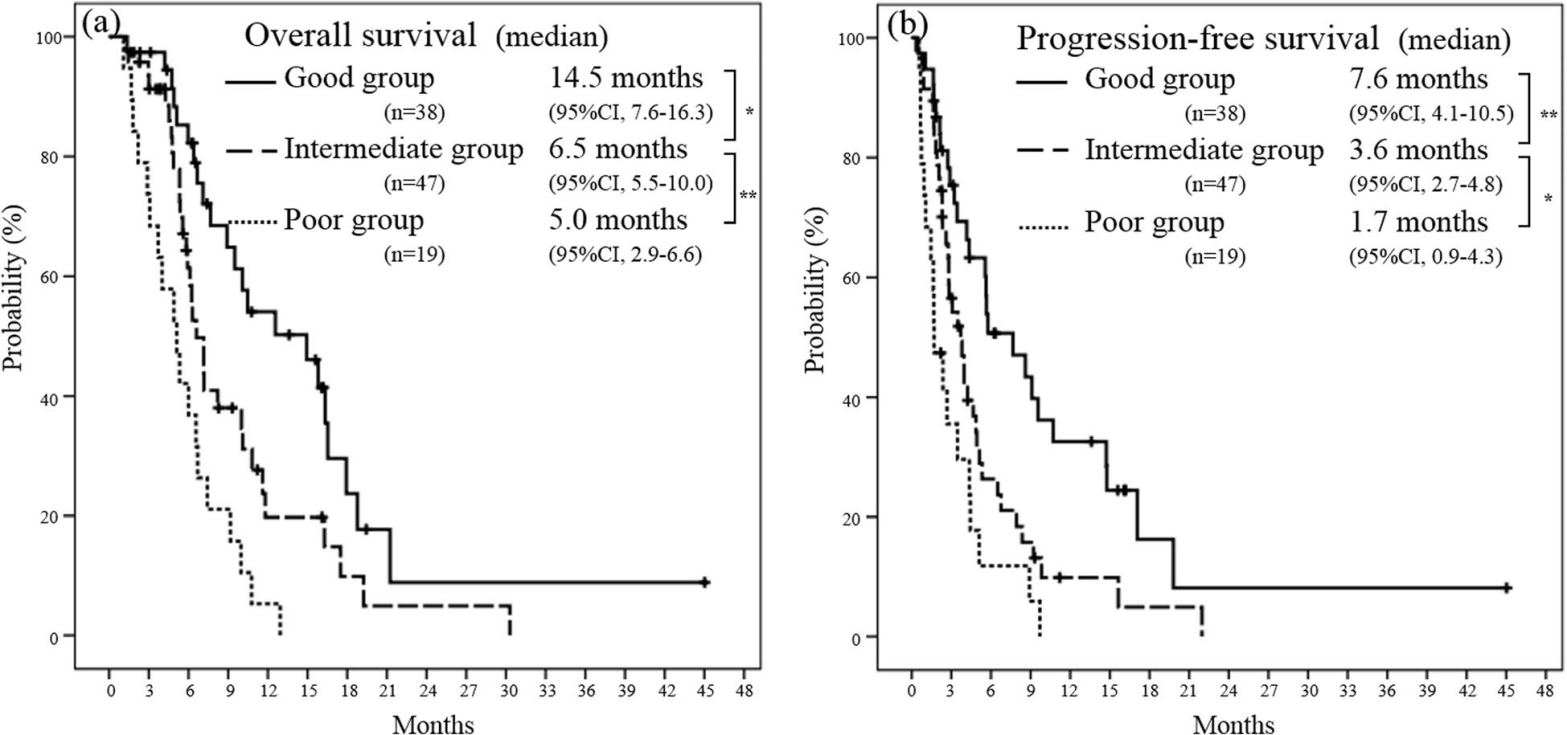
Kaplan-Meier analysis of (**a**) overall survival and (**b**) progression-free survival for three subgroups (the poor, intermediate, and good prognostic groups) according to prognostic index using Glasgow prognostic score and carcinoembryonic antigen level. *CI* confidence interval. *p < 0.05, **p < 0.01

The results of the Cox proportional hazards model are shown in Table 4. In the multivariate analysis, CEA level ≤ 10 ng/mL and GPS = 0 were identified as independent prognostic factors. For the clinical application of these findings, a prognostic index was calculated. One point was assigned for each variable and added for a composite score of 0–2. The patients were then assigned to three subgroups according to their prognostic index, as follows: good prognostic group, prognostic index = 0 (*n* = 38); intermediate prognostic group, prognostic index = 1 (*n* = 47); and poor prognostic group, prognostic index = 2 (*n* = 19). The median OS time was 14.7 months (95% CI 7.6–16.3) in the good prognostic group, 6.5 months (95% CI 5.5–10.0) in the intermediate prognostic group, and 5.0 months (95% CI 2.9–6.6) in the poor prognostic group (good vs intermediate, *p* < 0.05; intermediate vs poor, *p* < 0.01) (Fig. 2a). The median PFS time was 7.6 months (95% CI 4.1–10.5) in the good prognostic group, 3.6 months (95% CI 2.7–4.8) in the intermediate prognostic group, and 1.7 months (95% CI 0.9–4.3) in the poor prognostic group (good vs intermediate, *p* < 0.01; intermediate vs poor, *p* < 0.05) (Fig. 2b). Our prognostic model was able to classify the patients into three groups with significantly different values for PFS and OS.

**Table 4:** Multivariate analysis of prognostic factors

Variable	HR	95% CI	*p* value
GPS = 0	0.47	0.29–0.76	< 0.002
CEA ≤ 10 ng/mL	0.47	0.29–0.78	< 0.003

*CEA* carcinoembryonic antigen, *CI* confidence interval, *HR* hazard ratio, *GPS* Glasgow prognostic score

## Discussion

This retrospective study investigated mFFX therapy in patients with GnP-refractory MPC. The patients demonstrated an objective response rate of 10.6%, disease control rate of 56.7%, and median PFS and OS of 3.9 and 7.0 months, respectively. The median OS time after the start of the first-line therapy was 15.9 months. After exploring the independent variables associated with survival in this setting, we identified the CEA level and GPS as independent prognostic factors. This study also showed that the median PFS and OS of patients in good general condition with these good prognostic factors were 7.6 and 14.7 months, respectively.

Regarding second-line chemotherapy for GEM-refractory PC, a prematurely discontinued randomized controlled trial (RCT) by the German CONKO-study group provided evidence on the survival benefit of second-line chemotherapy compared with best supportive care [9]. In a recent NAPOLI-1 phase III trial, irinotecan liposomal injection combined with 5-FU/l-leucovorin significantly improved both of PFS and OS in patients with MPC after GEM-based therapy, showed a median PFS and OS of 3.1 and 6.1 months, respectively, and, indicating a new treatment option for this population [10]. Oxaliplatin combination therapy after GEM-based therapy has also been investigated by three RCTs, with confounding and different results (CONKO-003, SOX [S-1 plus oxaliplatin] and PANCREOX) [13, 14]. In a recent large phase III GRAPE trial comparing S-1 plus leucovorin and S-1 in patients with a good PS and Alb ≥3.5 g/dL, the median PFS and OS were 2.8 and 7.9 months, respectively [15]. Meanwhile, in our study, the median PFS and OS for patients with Alb ≥3.5 g/dL were 5.1 and 9.5 months, respectively. Sequential therapies with FFX and GnP have been tested because FFX and GnP are more effective as first-line therapy than GEM alone. A prospective cohort study in France evaluated second-line GnP after first-line FFX in 57 patients and showed a median PFS and OS of 5.1 months and 8.8 months, respectively, and a median OS of 18 months since the start of first-line FFX [16]. Although comparisons among these previous studies are difficult, their findings led us to suggest that mFFX in selected patients may be effective as a second-line treatment after failure of GnP.

As the FFX regimen has demonstrated better survival benefits than GEM as a first-line treatment, several prospective and retrospective studies evaluated FFX after failure of GEM-based chemotherapy, demonstrating median PFS time and OS times of 2.8–5.8 and 8.4–9.8 months [17–20]. These results suggest the promising clinical efficacy of the FFX regimen as a second-line treatment. Because FFX is a potentially highly toxic combination of drugs with serious side effects, two of the above studies, which are prospective phase II trial conducted by two Korean groups, reduced the dosage of both irinotecan (120–135 mg/m^2^) and oxaliplatin (60–65 mg/m^2^). Despite the dose reduction, these studies showed a promising efficacy of the regimen for patients who failed GEM-based chemotherapy. In these studies, most of the patients received GEM monotherapy or GEM plus erlotinib as a first-line treatment. In our study, we evaluated the efficacy and tolerability of mFFX after failure of first-line GnP and found the RDIs of oxaliplatin and irinotecan to be 80.0 and 77.2%, respectively. Prospective studies are needed to better define the doses of FFX and to determine the efficacy and toxicity of mFFX after failure of GnP.

Our study also found that grade 3–4 toxicities occurred in 57 patients (54.8%) and consisted mainly of hematological adverse effects (grade 3–4 neutropenia, 42.3%; febrile neutropenia, 5.8%). Although these incidences of hematologic toxicities are relatively higher than those reported for second-line chemotherapy regimens in previous studies such as the NAPOLI-1 and GRAPE trials and in the previous French cohort treated with GnP after FFX, they are comparable with those reported for mFFX as first-line chemotherapy in Japan. In our study, the treatment was generally well tolerated, and most episodes of the adverse events were reversible. Dose reduction and cycle delay were required in about 70% of the patients in this setting. Moreover, the RDI in this setting was relatively low compared with that of mFFX as a first-line treatment in a phase II study in Japan [5]. Although the safety profile in our retrospective study suggests that mFFX can be safe after failure of GnP, the dosage and treatment schedule of mFFX in this setting should be more carefully evaluated in the future. Indeed, several prospective studies to evaluate FFX after failure of GEM-based therapy have reduced the initial dose of irinotecan and oxaliplatin.

One of the major concerns with sequential therapies with GnP and mFFX is the risk of severe chronic PN owing to the treatment of nab-paclitaxel after oxaliplatin. In this study, the incidence of grade 1–2 PN was 78.8%, and that of grade 3–4 PN was 10.6%. This toxicity rate is higher than that of FFX or mFFX as first-line chemotherapy in Japan [5, 21]. However, this rate is consistent with that of FFX in the ACCORD11 trial or in the previous prospective French cohort treated with GnP after FFX, and is quite lower than that in the MPACT trial [3, 4, 16]. The mechanisms of nab-paclitaxel and oxaliplatin neuropathy are different, and so are their mechanisms of reversibility. Nab-paclitaxel causes paresthesia such as an abnormal sensation of the skin and distal burning pain, but these symptoms generally improve within 2 months after cessation of chemotherapy [22]. Oxaliplatin causes distal sensory decline and cumulative symmetric paresthesia [23]. Therefore, despite an equivalence in toxicity grade by both drugs, the neurotoxic impact of oxaliplatin after nab-paclitaxel on patients’ quality of life (QOL) may be less severe. This notion is supported by the finding that a treatment with a combination of nab-paclitaxel and FOLFOX did not develop significant neurotoxicity when they received fewer than 10 cycles, which is consistent with the occurrence of neuropathy in patients treated with FOLFOX alone [24]. As for nab-paclitaxel-induced PN, the MPACT trial revealed that the median time to improvement from grade 3 to grade 2 was 21 days and that from grade 3 to grade 1 or resolution of the event was 29 days. With a median of only 6 cycles of mFFX per patient in our study, the risk of severe neurotoxicity seems limited in this study.

Subgroup analysis of the patients in this study showed that a low GPS and a low CEA level were independently associated with a good outcome. Previous reports indicated that the PS, CA19–9 level, duration of the first-line chemotherapy, and systemic inflammation-based prognostic score were important prognostic factors in a salvage setting [11, 12, 25–29]. It is now widely accepted that the systemic inflammation-based prognostic score, such as the GPS, is one of the reliable indicators of survival for many types of malignant solid tumors including PC in various settings [11, 30]. Tumor markers such as CEA and CA19–9 levels were also reported as important prognostic factors in patients with advanced PC treated with chemotherapy. In our study, although the CA19–9 level and survival did not show a significant difference, patients in this setting with high CEA levels might indicate a more aggressive tumor biology. The question of whether the benefits of mFFX extend to patients even after failure of GnP is relevant because both mFFX and GnP are more intensive than GEM alone in the first-line setting and there is no established standard second-line chemotherapy regimen after failure of GnP. Our prognostic model using GPS and CEA levels classified the patients into three prognostic groups (good, intermediate, and poor) and enabled stratification of patients according to both PFS and OS. The median OS times in the poor, intermediate, and good prognostic group were 5.0, 6.5, and 14.7 months, respectively. These results suggest that mFFX after failure of GnP offered no survival benefits to the poor prognostic group. Thus, the model might be useful for deciding whether mFFX is indicated for the patients with MPC in this setting.

This study has a few limitations. First, we only performed a single-center retrospective analysis, although the sample size exceeded 100 patients. Second, the patients who received treatment might have been more fit, better able to tolerate the treatment, and therefore more likely to benefit from it. Additionally, the gap between the median PFS time and median OS time in the good prognostic group was relatively large. In the good prognostic group, 28% of patients received chemotherapy after failure of second-line mFFX. In a previous meta-analysis, post-progression survival following second-line chemotherapy in patients with advanced PC was associated with the rate of subsequent chemotherapy [31]. The subsequent treatment and bias from selecting patients with a good general condition may explain these findings. Lastly, this study did not evaluate biomarkers and QOL. QOL is an important element of palliative chemotherapy for PC. Hence, future prospective studies of second-line mFFX following GnP are necessary to clarify treatment efficacy and QOL of patients in this setting.

## Conclusions

mFFX showed to be a tolerable and effective second-line treatment for selected patients with MPC after failure of GnP therapy. Our prognostic model using GPS and CEA level might be useful for deciding whether mFFX is indicated in this setting. These findings need to be confirmed in a comparative RCT.

## Data Availability

All the data and materials supporting the conclusions were included in the main paper. The datasets used in the current study could be available from the corresponding author on request.

## Abbreviations

ADI: Actual dose intensity
Alb: Albumin
CA19–9: Carbohydrate antigen 19–9
CEA: Carcinoembryonic antigen
CI: Confidential interval
CRP: C reactive protein
CTCAE: Common terminology criteria of adverse events
ECOG: Eastern Cooperative Oncology Group
FFX: FOLFIRINOX
GEM: Gemcitabine
GnP: Gemcitabine plus nab-paclitaxel
GPS: Glasgow prognostic scale
mFFX: Modified FOLFIRINOX
MPC: Metastatic pancreatic cancer
NLR: Neutrophil-to-lymphocyte ratio
OS: Overall survival
PC: Pancreatic cancer
PFS: Progression free survival
PN: Peripheral neuropathy
PS: Performance status
RCT: Randomized controlled trial
RDI: Relative dose intensity
RECIST: Response Evaluation Criteria in Solid Tumors
SDI: Standard dose intensity
UGT1A1: Uridine diphosphate glucuronosyltransferase 1A1
